# Rare case of young cardiac amyloidosis: difficult diagnostic pathway in routine clinical care

**DOI:** 10.1093/ehjcr/ytag231

**Published:** 2026-03-23

**Authors:** Olga S Chumakova, Elena N Dankovtseva, Maria A Sannikova, Elena A Mershina, Svetlana A Alexandrova, Elena A Stepanova, Mariya Y Suvorina, Dmitry A Zateyshchikov

**Affiliations:** Department of Cardiology, Moscow Municipal Clinical Hospital #17, Volynskaya street, 7, Moscow 119620, Russia; Department of Cardiology, Moscow Municipal Clinical Hospital #29 Named After N. E. Bauman, Hospital squire, 2, Moscow 111020, Russia; Department of Hematology, Botkin Hospital, 2nd Botkinsky Proezd, 5, Moscow 125284, Russia; Medical Research and Education Center, Moscow State University Named After M.V. Lomonsov, Lomonosovsky Prospect, 2, Moscow 119991, Russia; Radiology Department, Bakulev National Medical Research Center for Cardiovascular Surgery, Rublevskoe highway 135, Moscow 121552, Russia; Department of Pathology, Russian Medical Academy of Continuous Professional Education, Barrikadnaya street, 2/1, Moscow 125993, Russia; Russian Academy of Sciences, Institute of Protein Research, Institutskaya street, 4, Pushchino, Moscow region 142290, Russia; Department of Cardiology, Moscow Municipal Clinical Hospital #29 Named After N. E. Bauman, Hospital squire, 2, Moscow 111020, Russia

**Keywords:** Light chain amyloidosis, Case report, Young, Hypertrophic cardiomyopathy, Phenocopy, Diagnostics

## Abstract

**Background:**

Light-chain amyloidosis (AL) is a rare plasma cell disorder characterized by extracellular deposition of misfolded light chains in multiple organs, typically manifesting with non-specific symptoms that result in delayed diagnosis. Cardiac involvement is a major adverse prognostic factor. The incidence peaks around the age of 65, and occurrence in younger individuals is exceptionally rare, further complicating timely recognition.

**Case summary:**

We report a 41-year-old female patient who exhibited a 1-year course of progressive heart failure, ultimately diagnosed as AL amyloidosis. The ‘red flags’ were either overlooked or misattributed to other causes of left ventricular hypertrophy, including hypertrophic cardiomyopathy and Fabry disease, partly due to the patient’s atypically young age and family history. The initial suspicion of cardiac amyloidosis was based on advanced cardiac magnetic resonance imaging, which was not immediately available at the time. The initial tissue biopsy result was negative, necessitating an expert re-evaluation with a polarized light microscopy. Laboratory workup for AL amyloidosis revealed non-IgM monoclonal gammopathy of undetermined significance as a preceded plasma cell disorder.

**Discussion:**

The diagnosis of AL amyloidosis requires evidence of plasma cell dyscrasia through serum/urine immunochemistry, in addition to the detection and typing of amyloid in tissues. Given the rapid progression of the disease and the poor outcomes observed in the absence of timely targeted therapy, broader laboratory screening for AL amyloidosis should be considered in patients with unexplained hypertrophic phenotype or heart failure, irrespective of age. The improved access to expert multidisciplinary teams through dedicated cardiomyopathy centres is warranted.

Learning points

- Consider AL cardiac amyloidosis in any patient with unexplained left ventricular hypertrophy and clinical ‘red flags,’ regardless of age.
- Prompt laboratory screening (serum free light chain assay and serum/urine immunofixation) followed by confirmatory biopsy is essential, as delayed diagnosis in rapidly progressive heart failure significantly worsens outcomes.

## Introduction

Systemic amyloid diseases are rare protein-folding disorders characterized by extracellular deposition of misfolded autologous proteins as β-sheet fibrils. These fibrils infiltrate multiple organs, producing indistinct symptoms and making amyloidosis a persistent diagnostic challenge.^1^ The precursor protein determines organ tropism and guides therapeutic strategy.

Cardiac involvement typically manifests as increased left ventricular (LV) wall thickness, mimicking other causes of LV hypertrophy, particularly hypertrophic cardiomyopathy (HCM). Over 98% of cardiac cases arise from monoclonal immunoglobulin light chains (AL amyloidosis) or transthyretin (ATTR amyloidosis), the latter encompasses both wild-type and hereditary forms.^2^

In AL amyloidosis, misfolded free light chains (FLC) originate from a small, slowly proliferating clone of plasma cells in the bone marrow. This condition is frequently preceded by other plasma cell disorders, including monoclonal gammopathy of undetermined significance (MGUS) or multiple myeloma.^3^ The heart and kidneys are most frequently affected (in around 50–75% of cases), followed by the peripheral nerves, liver, gastrointestinal tract, and soft tissues.^3^ The incidence of AL amyloidosis is low, at 1.0–1.7 per 100 000 person-years,^4,5^ with an average age at diagnosis of 65 years.^3,5^

Despite the significant survival benefits afforded by emerging therapies in AL amyloidosis,^6,7^ the prognosis remains poor in cases involving the heart. Median survival is a mere 1.5 years, and there is a precipitous decline after the onset of symptomatic HF.^8,9^ Early-stage diagnosis is the only chance for prolonged survival; however, diagnosis is typically delayed by an average of 10 months due to non-specific symptoms, low clinical suspicion, and limitations in the healthcare system.^10^

**Figure 1:**
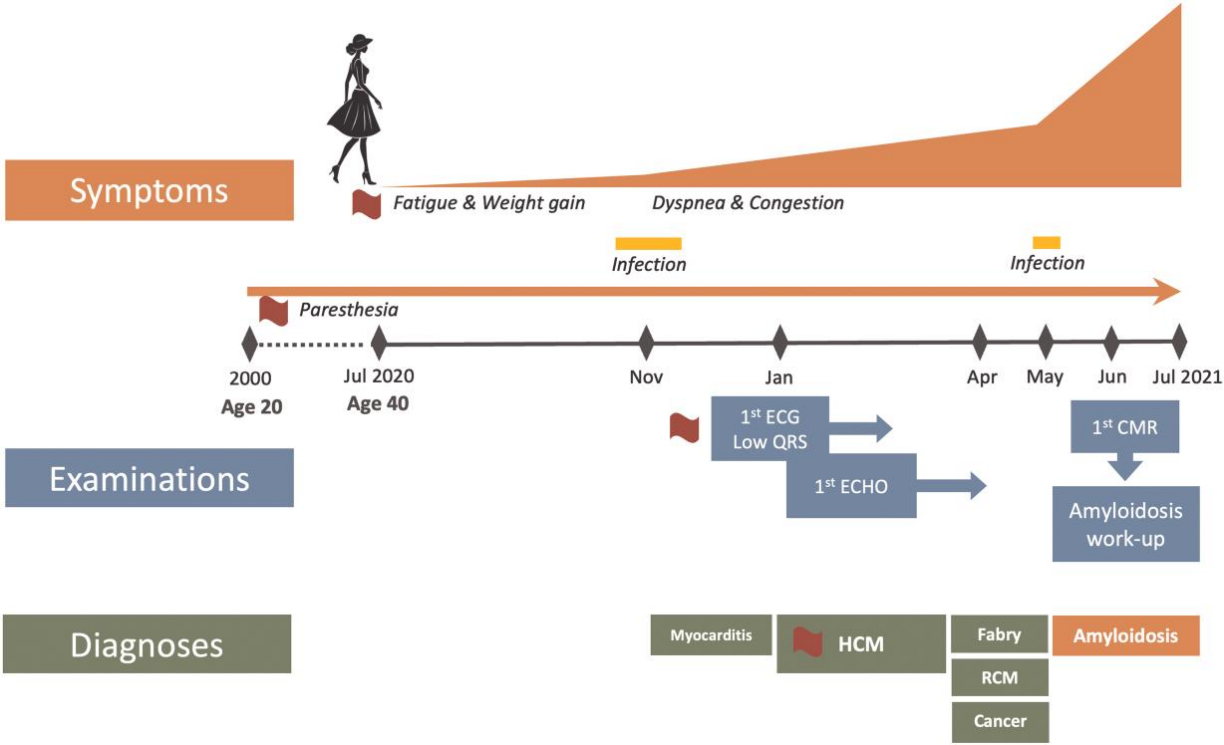
Timeline.

In this study, we present a challenging case of cardiac AL amyloidosis in a young female patient who was initially misdiagnosed with HCM. Atypical demographics, in conjunction with the aforementioned contributing factors, further delayed diagnosis (*Figure 1*). This report seeks to raise awareness, highlight diagnostic pitfalls, and emphasize the importance of a multidisciplinary approach involving experts for the timely initiation of targeted therapy.

## Summary figure

**Figure:**
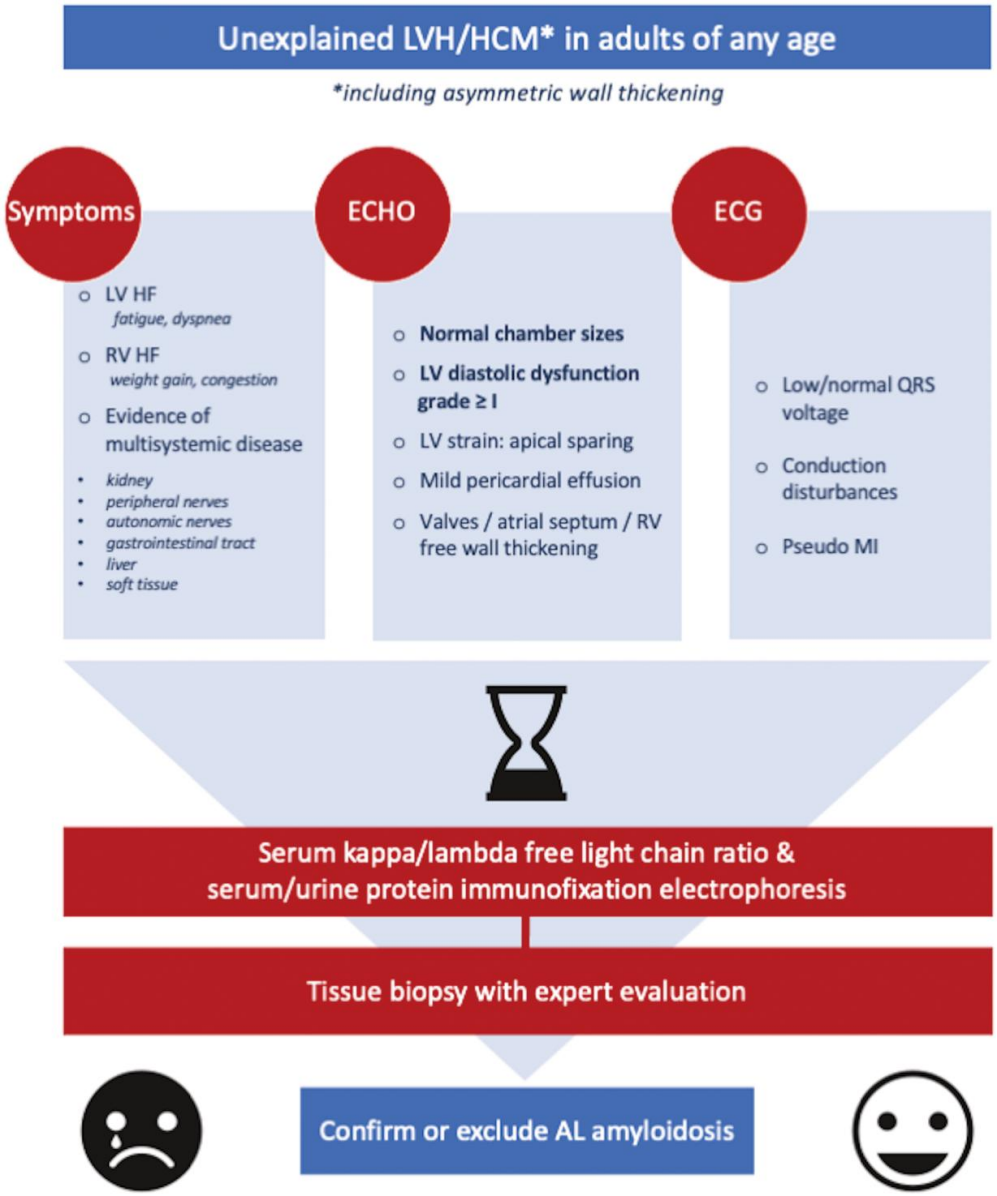
The diagram summarizes key clinical, echocardiographic, and electrocardiographic ‘red flags’ of cardiac AL amyloidosis that should prompt laboratory testing to confirm or exclude the diagnosis without delay.

## Case presentation

**Figure 2:**
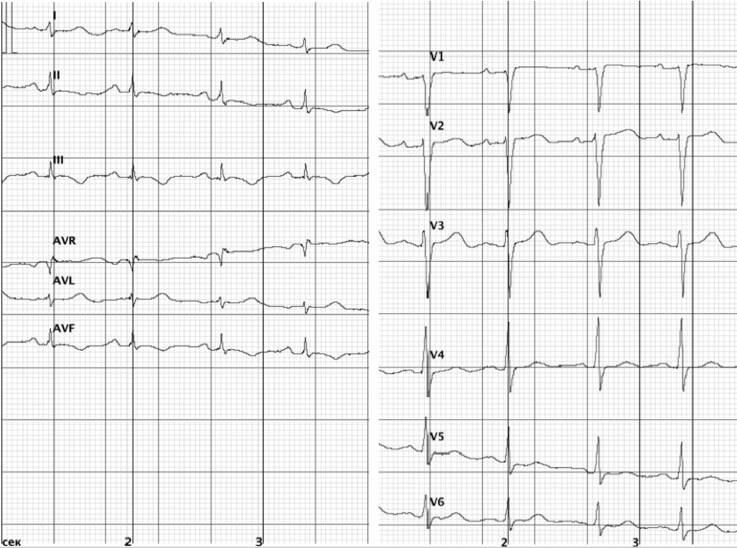
Electrocardiogram shows normal sinus rhythm, low QRS voltage in the limb leads, ‘normal’ QRS voltage in the precordial leads, R-wave regression in V2, and moderate inferolateral repolarization abnormalities with flat to 1 mm inverted T-waves.

A 41-year-old female patient was admitted to hospital with palpitations, exertional dyspnoea, and leg oedema. The onset of her condition occurred 9 months earlier manifesting as fatigue and weight gain. Four months later, during a respiratory infection, new symptoms of exertional dyspnoea and mild leg oedema appeared, prompting her first visit to a general practitioner. Myocarditis was suspected; however, none of the confirmatory tests, including cardiac imaging, were documented. Two months later, the persistent symptoms prompted her to seek a cardiology evaluation, which revealed stable vital signs and trace leg oedema. Echocardiogram showed interventricular septal thickness of 17 mm, posterior wall thickness of 14 mm, LV ejection fraction (EF) of 73%, and no other abnormalities. Electrocardiogram (ECG) demonstrated sinus rhythm, low-to-normal QRS voltage, and inferolateral repolarization changes (*Figure 2*). Routine laboratory tests, encompassing parameters of haematology, creatinine, liver transaminases, potassium, glucose, and thyroid function, were normal. The diagnosis was nonobstructive HCM, and treatment with a beta-blocker and a thiazide diuretic was initiated for New York Heart Association (NYHA) Class II HF. Despite the therapy, dyspnoea progressed to NYHA Class III over the subsequent 3 months, necessitating hospital admission. The patient’s personal history included temperature intolerance and hand paraesthesia since the age of 20, as well as chronic calculous cholecystitis.

**Figure 3:**
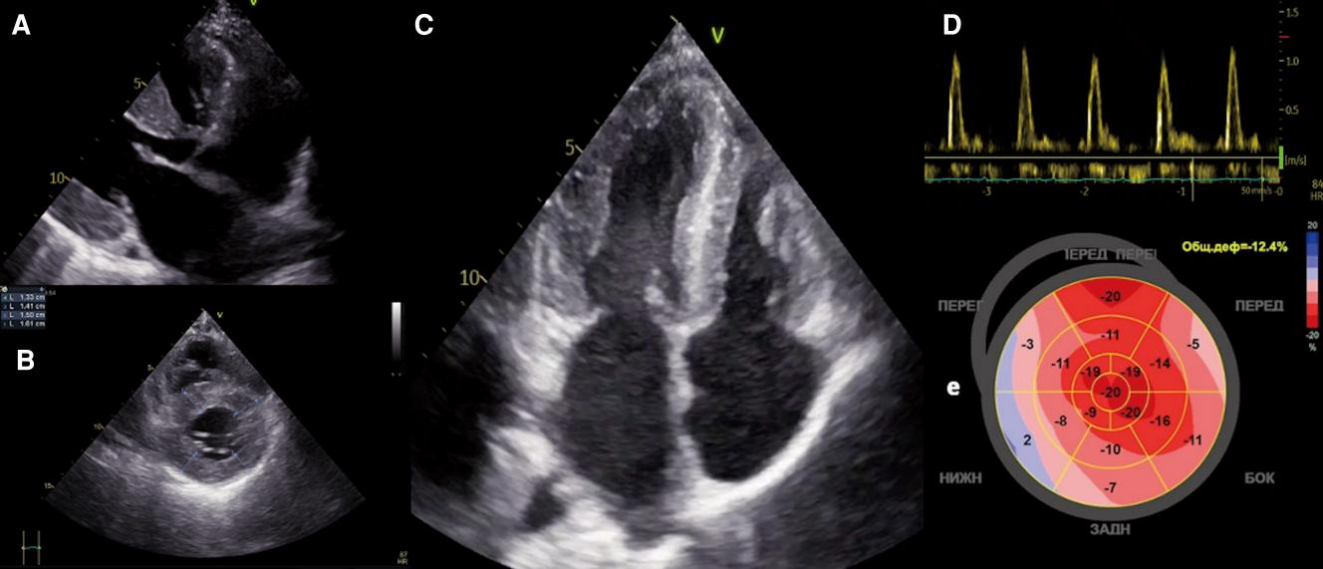
Echocardiogram shows (*A–B*) concentric LV hypertrophy with a maximal wall thickness of 15 mm, (*C*) reduced LV end-diastolic volume (25 mL/m^2^) and mild left atrial enlargement (35 mL/m^2^), (*D*) markedly elevated E/A ratio on mitral inflow Doppler (4.7), and (*E*) decreased global longitudinal strain (12.4%) with a ‘cherry on top’ pattern.

On examination, the patient had moderate peripheral oedema, blood pressure of 110/80 mmHg, heart rate (HR) of 80 bpm, and body mass index of 21.2 kg/m^2^. Thoracic computed tomography and abdominal ultrasound scans were unremarkable. ECG showed the same abnormalities as 3three months ago. Echocardiogram revealed concentric LV hypertrophy with maximal wall thickness of 15 mm, borderline LV EF (48%), restrictive mitral inflow pattern (E/A—4.7), elevated E/e′ ratio (19.6), mild left atrial enlargement (35 mL/m^2^), and moderate elevation of systolic pulmonary artery pressure (43 mmHg) (*Figure 3A–3D*). Strain and cardiac magnetic resonance (CMR) were not available. Holter monitoring revealed sinus rhythm (average HR 89 bpm), brief episodes of bradycardia (40 bpm), and supraventricular tachycardia (177 bpm), and two short runs (seven beats) of non-sustained ventricular tachycardia (NSVT). Laboratory tests indicated moderate renal impairment (serum creatinine 125 µmol/L, estimated glomerular filtration rate (eGFR) 48 mL/min/1.73m^2^, proteinuria 0.13 g/L), and slightly raised C-reactive protein (CRP) (8.6 mg/L). N-terminal pro-brain natriuretic peptide (NT-proBNP) was ordered, but the test was not performed. The thyroid and ophthalmological examinations yielded normal results. Restrictive cardiomyopathy—a HCM phenocopy—was suspected, with Fabry disease being a particular consideration. Parents’ cardiac examinations were unremarkable. However, the mother reported a history of similar neurological symptoms that had been present since her forties. Dried blood spots for lysosomal storage disorders (metabolite levels and *GLA* gene) were dispatched for analysis. Treatment with bisoprolol, spironolactone, and torasemide resulted in an improvement in symptoms within a week. The patient was discharged with a referral for expert CMR and a follow-up appointment.

Two weeks later, she was admitted to a different hospital with a fever and abdominal pain. Acute calculous cholecystitis and COVID-19 pneumonia were diagnosed; ultrasound further revealed hepatomegaly and mild ascites. Following a week of antibacterial and anti-inflammatory treatment, the patient exhibited signs of improvement and was discharged. The normal results in lysosomal metabolite analysis made Fabry disease less probable. CMR was still pending due to insurance and financial barriers.

Three weeks later, the patient was readmitted to the hospital with worsening HF—severe leg oedema, abdominal distension, and dyspnoea at rest; her weight had increased by 3 kilos; vitals were stable. A repeat echocardiography showed severe tricuspid regurgitation and right atrial enlargement (46 mL/m^2^), along with mild pericardial effusion; the dimensions of the left-sided chambers and ventricular wall thickness remained unchanged compared with the previous examination. Chest X-ray revealed mild pleural effusion; ultrasound identified moderate hepatomegaly and diffuse renal changes suggestive of oedema and infiltration secondary to HF. Laboratory tests indicated severe renal impairment (serum creatinine 273 µmol/L, eGFR 19 mL/min/1.73m^2^, proteinuria 1.5 g/day), markedly elevated serum NT-proBNP (>35000 pg/mL), hyperuricaemia (813 µmol/L), mild anaemia (107 g/L), hypoalbuminemia (32 g/L), elevated D-dimer, and slightly raised CRP (11 mg/L); lactate dehydrogenase and ferritin were normal, ruling out hemochromatosis. Fabry disease was excluded on the basis of a negative *GLA* mutation test. The Holter monitor showed sinus rhythm (average HR 71 bpm), brief episodes of atrioventricular nodal rhythm (up to nine beats), and six NSVT runs (the longest lasting 27 s). Mild conduction disturbances were attributed to the beta-blocker, resulting in a reduction of bisoprolol dosage by half.

**Figure 4:**
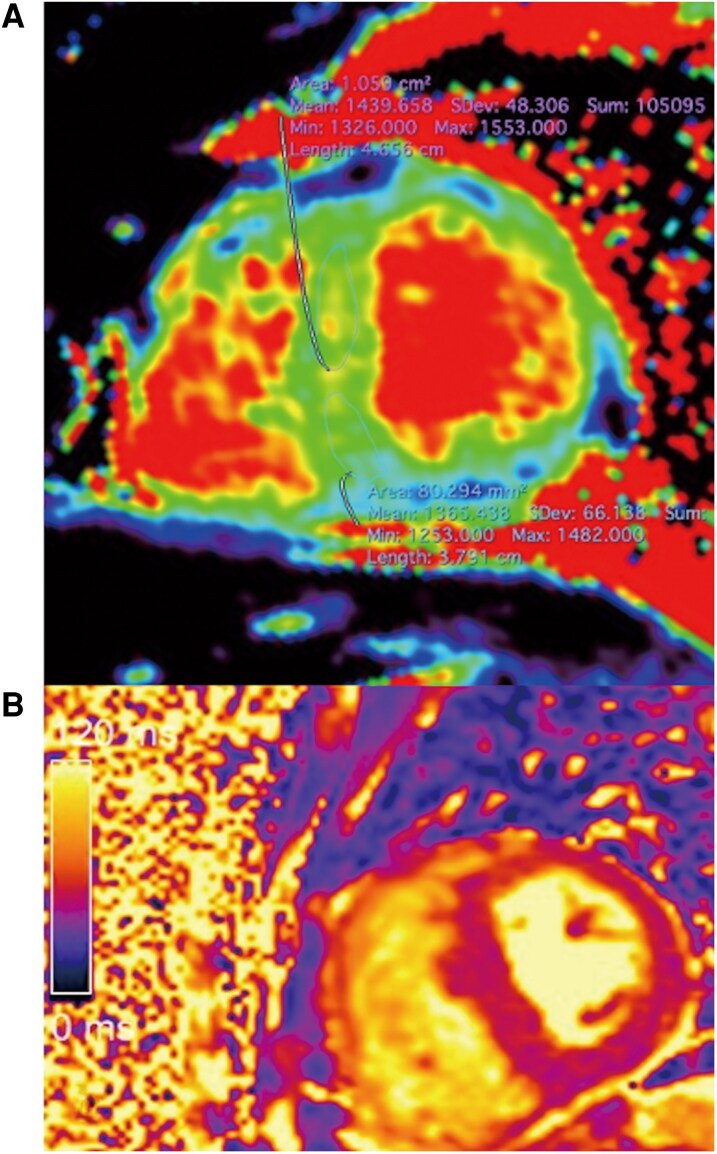
Non-contrast CMR imaging at 1.5 T: (*A*) Elevated native T1 relaxation times, with a maximum of 1365 ms in the infero-septal segment (*N* = 995 ± 32) and 1439 ms in the antero-septal segment (*N* = 979 ± 39); (*B*) T2 relaxation times of 59 ms, at the upper limit of normal (55 ± 5), with no signs of myocardial oedema.

**Figure 5:**
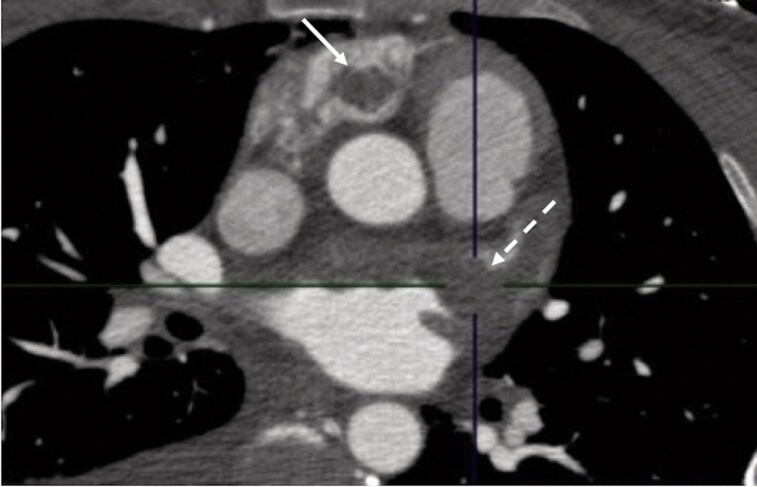
Contrast-enhanced CT. Solid arrow: thrombus in the right atrial appendage; dotted arrow: thrombus in the left atrial appendage.

Initial non-contrast CMR (1.5 T) performed at another institution confirmed echocardiographic abnormalities and revealed diffusely increased native T1 values (>1200 msec), suggestive of amyloidosis (*Figure 4*). It also showed round mass-like formations in both atrial appendages, seen in the absence of atrial fibrillation, raising suspicion for rare malignancy such as myofibroblastic tumour or cardiac lymphoma. Subsequent contrast-enhanced computed tomography excluded the malignancy and identified the masses as thrombi (*Figure 5*). Normal serum total protein and calcium levels, as well as the absence of brain or bone lesions, provided compelling evidence that multiple myeloma was not the underlying cause of the patient’s symptoms.

In the following 10-day period, the systemic amyloidosis workup was conducted. Serum and daily urine samples were dispatched to a specialist haematology centre for electrophoresis, immunofixation, and FLC assay. Given the patient’s young age, wild-type ATTR amyloidosis was deemed unlikely; therefore, bone scintigraphy with bisphosphonates, which would have required transfer to another clinic, was not performed; however, dried blood spots were sent for *TTR* gene analysis. To minimize the risk to the patient, the surgical fat biopsy from the abdominal wall was prioritized over the more invasive direct cardiac or renal biopsy. To expedite the process, sternal bone marrow aspiration was completed prior to the availability of the immunochemistry results.

**Figure 6:**
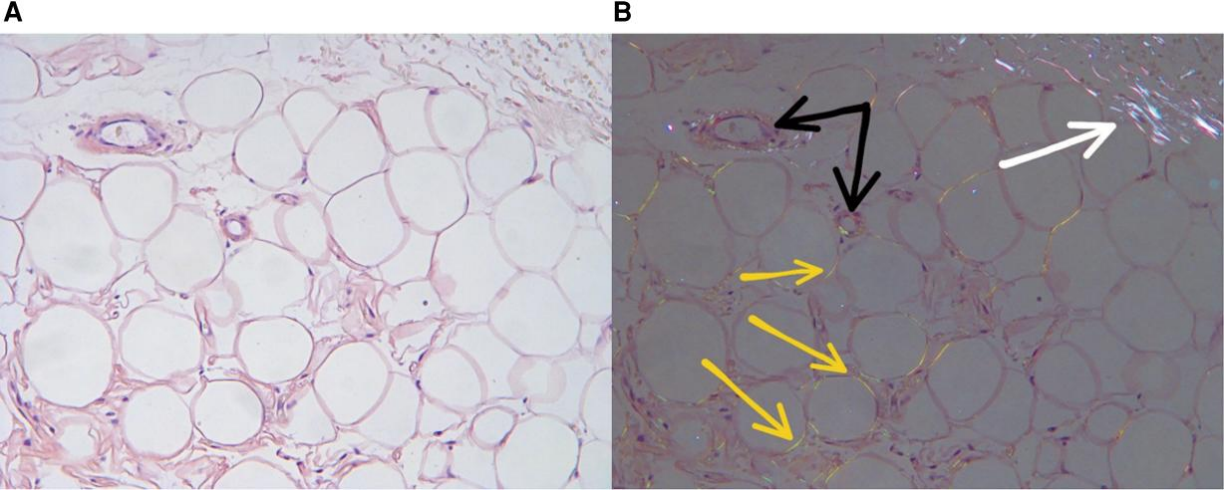
Congo red staining of subcutaneous adipose tissue: (*A*) bright-field microscopy; (*B*) polarized light microscopy of the same field. Numerous amyloid deposits in the walls of microvasculature vessels (black arrows) and along adipocyte membranes (yellow arrows); collagen fibers (white arrow) with white birefringence, in contrast to the amyloid typical birefringence. Sections prepared from paraffin blocks; magnification 400x.

**Figure 7:**
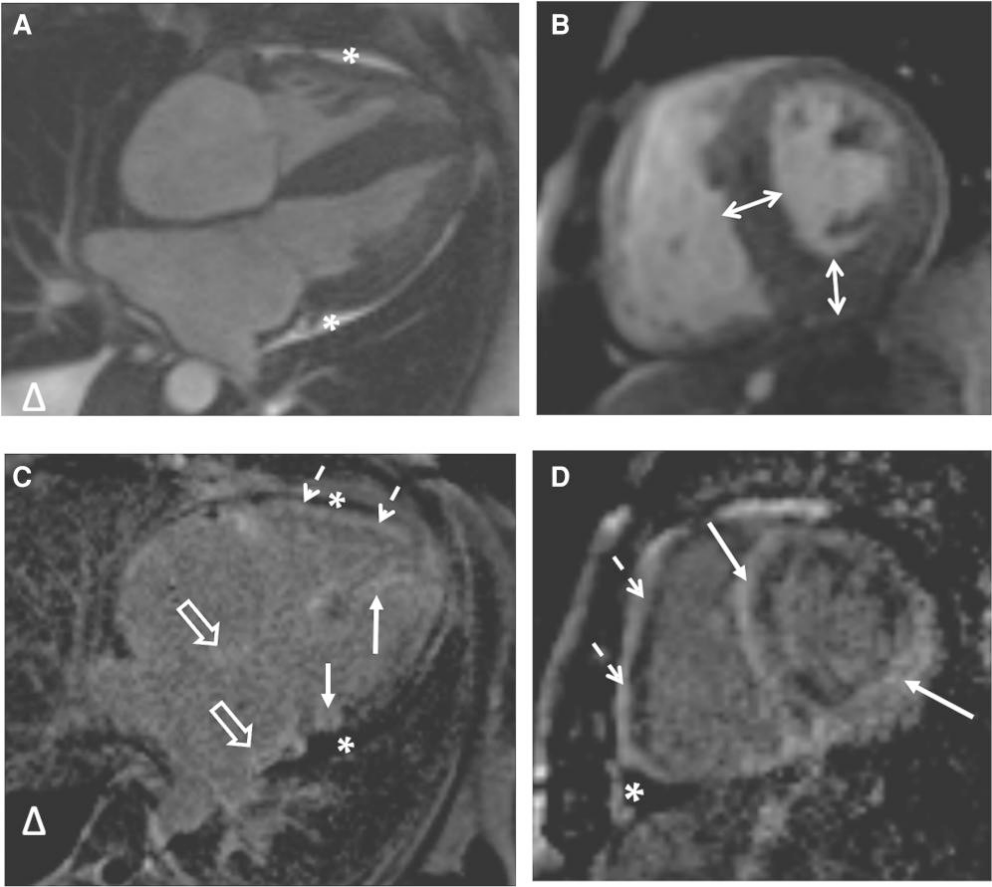
Contrast CMR imaging: cine long- (*A*) and short-axis (*B*) images (SSFP-sequence). Asymmetric LV hypertrophy with interventricular septal and inferior wall thickness of 14–15 mm (double-ended arrows); right-sided hydrothorax (triangle) and small pericardial effusion (asterisk) are present. LGE images (*C–D*), acquired at the same positions (PSIR-sequence), show extensive enhancement in the right (dotted arrows) and left ventricular walls (solid arrows), as well as in the atrial walls (empty arrows).

The Congo red staining of the fat biopsy under normal light in the local laboratory revealed no amyloid deposits. *TTR* gene testing was negative, thereby ruling out the inherited ATTR amyloidosis. A myelogram showed 8% plasma cells (normal 0.1–1.8), consistent with a plasma cell dyscrasia. Subsequent serum immunofixation and FLC assay revealed monoclonal A-lambda of 19.3 g/L (28% of total protein), FLC lambda of 1460 mg/L (normal 5.7–26.3), and a reduced kappa/lambda FLC ratio of 0.2 (normal 1.1–2.9). Urinary immunofixation detected the Bence Jones protein lambda at a rate of 0.82 g/24 h. Secondary hypogammaglobulinemia and elevated levels of *β2*—microglobulin were also present. Given that serum monoclonal protein was <30 g/L, marrow plasma cells were <10%, and multiple myeloma criteria was absent, the non-IgM MGUS was the most appropriate diagnosis. Expert re-examination of the fat biopsy under polarized light demonstrated amyloid deposits, including in the vessel walls, indicating systemic involvement (*Figure 6*). Mass spectrometry characterized the deposits as lambda light chain amyloid. Subsequent to the enhancement of renal function, contrast-enhanced CMR was performed at another centre of expertise. This revealed extensive, diffuse late gadolinium enhancement (LGE) in the myocardium of both ventricles, atrial walls, and atrioventricular valves; it also showed pleural and pericardial effusions and thrombi in both atrial appendages. These findings were strongly supportive of cardiac amyloidosis (*Figure 7*). At that moment, the HF management included bisoprolol, spironolactone, acetazolamide, and intravenous furosemide; in addition, temporary dopamine and saline were administered for hypotension, and unfractionated heparin for intracardiac thrombosis.

Three months after the initial admission, the patient was transferred to the haematology department with a diagnosis of non-IgM MGUS and systemic AL amyloidosis involving the heart (Stage IIIb, Mayo 2004 with European modifications), kidneys, and skin. ECG showed atrial fibrillation and complete right bundle branch block. Cyclophosphamide, bortezomib, and dexamethasone were initiated, but discontinuation was necessary on Day 4 due to rapid deterioration. The final echocardiogram revealed LV EF 35%, septal thickness of 19 mm, severe pericardial effusion, and severe pulmonary hypertension. Unfortunately, the patient died shortly thereafter; an autopsy was declined. One year later, reassessment of the initial echocardiogram showed impaired global longitudinal strain of −12.4% with preserved apical magnitude (*Figure 3E*).

## Discussion

AL amyloidosis in patients under the age of 40 is extremely rare condition, yet it exhibits the same negative prognostic markers as its older patient counterpart: Cardiac involvement, HF stage, and diagnostic delay.^11,12^

In our case, the disease manifested initially with fatigue, the most prevalent early symptom of AL amyloidosis (in around 80% of cases), which patients often misinterpret, thereby delaying medical attention.^10^ At the initial presentation, coincident symptoms of flu-like illness and HF signs suggested the myocarditis. However, confirmatory tests were not performed, likely due to clinical stability, mild symptomology, and COVID-19-related healthcare overload, which further prolonged the diagnosis.

HCM was the first confirmed diagnosis made 6 months after the onset of symptoms. Current guidelines advocate the contrast-enhanced CMR and genetic testing in all newly diagnosed HCM cases, given their high yield for alternative diagnoses and sudden cardiac death risk stratification. In our case, CMR was postponed due to constraints in resources, and was only considered after HF progression; genetic testing was omitted due to insurance barriers. Sarcomeric HCM patients frequently remain asymptomatic for years, with congestive HF signs appearing in <10% only at the burnout stage. Conversely, our patient developed symptomatic HF early, which ought to have raised the suspicion of a phenocopy. In this context, the ‘red flags’—low or ‘normal’ voltage on ECG in a patient with LV hypertrophy on cardiac imaging and peripheral neuropathy—would likely not have been overlooked.

At the patient’s initial admission, the signs of amyloidosis became more apparent. The patient exhibited symptoms of worsening congestion and new-onset renal impairment, and the low-to-normal QRS voltage on ECG was present but once again overlooked. It is noteworthy that she did not initially report symptoms of neuropathy, as these were mild and only became apparent upon direct questioning. However, the patient’s youth and family history of neuropathy shifted suspicion toward inherited phenocopies, which are known progress more slowly. Fabry disease is the most common genetic condition that present with a clinical profile similar to that of HCM in adults. Similarly to amyloidosis, it often involves the kidneys and peripheral nerves. In female subjects, lysosomal enzyme activity may be within the normal range; therefore, a diagnosis requires genetic confirmation, which in this case took 2 months.

CMR was chosen as the first-line investigation, being appropriate for the most cardiomyopathies. However, it is suboptimal for suspected amyloidosis in cases where advanced imaging is not promptly available. In our case, plasma cell dyscrasia testing was ordered only after the CMR suggested amyloidosis; consequently, the diagnosis was made 6 months after the initial medical consultation and 2 months after the onset of overt congestion. This aligns with global trends, where the median NYHA class at diagnosis is III,^11^ and one-third of patients present with congestive HF.^13^ Furthermore, even after suspicion arises from echocardiography or CMR, confirmatory testing typically takes 1–2 months.^10^ In our patient, chemotherapy was initiated a mere 1 month after CMR had indicated cardiac amyloidosis. Among the confirmatory tests, there is an assessment of cardiac uptake of bisphosphonates on bone scintigraphy. A positive result of grade 2 or 3, along with negative immunochemistry for AL amyloidosis, facilitates a non-invasive diagnosis of ATTR amyloidosis.^2^ However, biopsy is required where results are uncertain or inconsistent with the clinical course, as there is a possibility of false-positive and false-negative findings.

Large atrial appendage masses on initial CMR in the absence of atrial fibrillation were indicative of a potential malignancy, which was later excluded. In amyloidosis, the occurrence of intracardiac thrombi is possible even in cases of sinus rhythm, likely due to atrial wall amyloid infiltration, mechanical standstill, and blood stasis.^14^ The question of whether such patients benefit from prophylactic anticoagulation warrants further study. Our patient had a markedly diminished mitral inflow A-wave, accompanied by a modestly enlarged left atrium. This finding was more suggestive of atrial amyloid infiltration than of restrictive LV diastole.

Another obstacle was the initially negative result of the abdominal fat biopsy, despite the fact that this examination has a sensitivity of >80% for AL amyloidosis.^15^ This underscores the importance of expert pathology and polarized light microscopy in identifying amyloid deposits.

The patient’s peripheral neuropathy preceded cardiac symptoms by 20 years, which may be indicative of long-standing MGUS^16^ that progressed to AL amyloidosis. The prevalence of MGUS is low (<0.3%) in individuals under the age of 50,^17^ but the risk of progression is similar to that observed in older patients.^18^ Non-IgM MGUS with an abnormal serum FLC ratio and a large monoclonal protein size, as observed in our patient, carries a 30% risk of malignant transformation after 20 years.^19^ The aetiology of the condition remains unclear, although genetic factors may play a role.^20^ The patient’s mother, who exhibits a comparable neuropathy but lacks cardiac symptoms, may also have undiagnosed MGUS, necessitating subsequent monitoring.

From the outset, our patient fit a poor-risk profile: cardiac-dominant disease and a markedly elevated pretreatment difference between involved (lambda) and uninvolved (kappa) FLC justified for a high tumour burden.^8^ Additionally, two inflammatory episodes preceded disease progression, and although inflammation is not considered to be implicated in AL amyloidosis pathogenesis, elevated CRP predicts higher mortality in HF presence.^9^ Nevertheless, earlier initiation of bortezomib-based therapy had the potential to lead to a better response.

The management of severe heart failure in cardiac amyloidosis poses a significant challenge due to LV restrictive physiology, which makes cardiac output highly preload dependent. Diuretics must be carefully titrated to avoid hypovolemia, hypotension, and subsequent hypoperfusion. In our case, the patient was prescribed temporary nonstandard therapy—dopamine infusion—due to severe hypotension that developed during diuretic treatment.

In summary, in this young patient presented with unexplained LV hypertrophy and HF, the ‘red flags’ for amyloidosis were overlooked or misattributed; workups for other conditions were pursued, including CMR, but not early laboratory testing for AL amyloidosis; resource limitations further delayed initiation of targeted therapy, which failed to improve the prognosis.

## Conclusions

Early recognition of cardiac AL amyloidosis requires high clinical suspicion, even in younger patients with unexplained hypertrophic phenotype or HF. Given the disease’s poor prognosis, laboratory screening for monoclonal components should be an early diagnostic step (*Summary Figure*). The timely diagnosis and initiation of targeted therapy, amongst other factors, are dependent on access to expert multidisciplinary teams, thereby underscoring the need for dedicated cardiomyopathy centres.

## Supplementary Material

ytag231_Supplementary_Data

## Data Availability

The data underlying this paper will be shared on reasonable request to the corresponding author.

## Lead author biography

Olga Chumakova has worked as a cardiologist in intensive cardiac unit and cardiological department since 2006. Since 2016 as an accomplished researcher her special interest is non-ischaemic cardiomyopathies, including personal involvement in cardiac imaging and cardio genetics. Now evolving as an international researcher to achieve higher results in scientific work and disseminate knowledge to a wider audience.

## Notes

**Consent:** The authors confirm that consent for the use of data from medical records, including images, for publication has been obtained from the patient in line with COPE guidance.
